# Molecular Diode Studies Based on a Highly Sensitive Molecular Measurement Technique

**DOI:** 10.3390/s17050956

**Published:** 2017-04-26

**Authors:** Madoka Iwane, Shintaro Fujii, Manabu Kiguchi

**Affiliations:** Department of Chemistry, Tokyo Institute of Technology, 2-12-1 Ookayama, Meguro-ku, Tokyo 152-8551, Japan; iwane.m.aa@m.titech.ac.jp

**Keywords:** molecular junction, single-molecule junction, molecular diode, electron transport

## Abstract

In 1974, molecular electronics pioneers Mark Ratner and Arieh Aviram predicted that a single molecule could act as a diode, in which electronic current can be rectified. The electronic rectification property of the diode is one of basic functions of electronic components and since then, the molecular diode has been investigated as a first single-molecule device that would have a practical application. In this review, we first describe the experimental fabrication and electronic characterization techniques of molecular diodes consisting of a small number of molecules or a single molecule. Then, two main mechanisms of the rectification property of the molecular diode are discussed. Finally, representative results for the molecular diode are reviewed and a brief outlook on crucial issues that need to be addressed in future research is discussed.

## 1 Introduction

**Figure 1:**
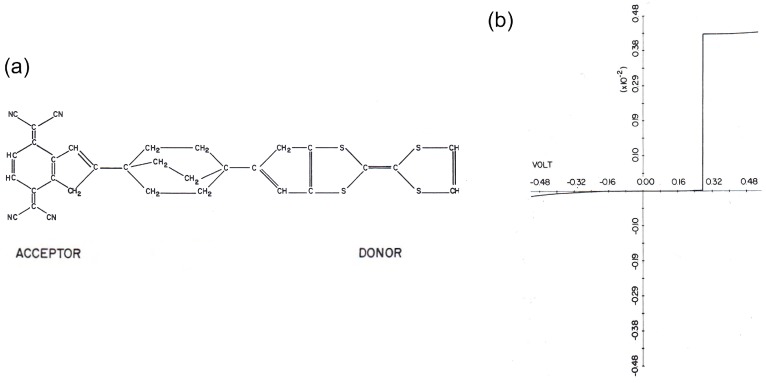
(**a**) Chemical structure of the rectifier molecule, acceptor tetracyanoquinodimethane (TCNQ) and the donor tetrathiofulvalene (TTF) are connected by a triple methylene bridge; (**b**) *I–V* characteristics of a rectifier molecule connected to two metal electrodes [11].

The diode is one of the basic components of electric devices. The diode acts as a rectifier, permitting electronic current to flow only in one direction. Rectification of electronic current was first achieved in a vacuum diode, then later in junctions where a p-type semiconductor is contacted with an n-type semiconductor [1]. Rectification occurs at a metal-semiconductor interface when a Schottky barrier is formed at the boundary between phases with different work functions or within a depletion layer. The rectifiers are commonly used in modern power supplies for electronics and the diodes are crucial for a broad range of applications from radios, logic gates, light emitting devices and photodetectors. For semiconductor junctions, the electronic properties including diode properties can be tuned by controlling the chemical species of dopants and their concentration in the semiconductors. Meanwhile, the electronic properties of molecular junctions can be controlled by designing the molecule based on advanced synthetic techniques [2,3,4,5,6,7]. Molecular diversity is one of the advantages of molecular electronics compared with the current Si-based electronics. At present, molecular devices such as diodes, transistors, switches and sensors, have been reported using functional organic molecules [8,9,10]. Among the molecular devices, the molecular diode is one of the major research targets in the field of molecular electronics. The investigation of the molecular diode was started in 1974 by Aviram and Ratner [11]. This was the first study of molecular electronics. They theoretically studied the electron transport through a model molecular junction (Figure 1a); the molecular backbone consists of donor and acceptor units connected by an insulating unit (triple methylene bridge). Figure 1b shows the calculated current-voltage characteristic of the molecular junction. The large current flows at positive bias, indicating that electrons preferentially flow from acceptor to donor units.

The molecular diode was first prepared by Langmuir-Blodgett (LB) technique where molecularly thin LB films were sandwiched by top and bottom metal electrodes [12] and the current-voltage characteristic with a rectification ratio of ~40 was reported for the LB-based molecular junction. In the last two decades, electronic rectification properties of the molecular diodes have been intensively investigated. Currently, an electronic rectification ratio of 1000 has been achieved for a molecular junction that is composed of molecular assemblies in a self-assembled monolayer (SAM) [13]. Around the year 2000, single-molecule measurement techniques were developed by several groups [14,15,16]. The rectification properties of the molecular junctions have been investigated on the single-molecule scale using these single-molecule measurement techniques. In this review, we firstly describe the fabrication technique of the molecular junctions and the measurement technique of the rectification properties of the molecular diodes consisting of the small number of molecules or a single molecule, and secondly the mechanisms of the rectification properties. Finally, representative works on the molecular diode are reviewed.

## 2. Molecular Junction

**Figure 2:**
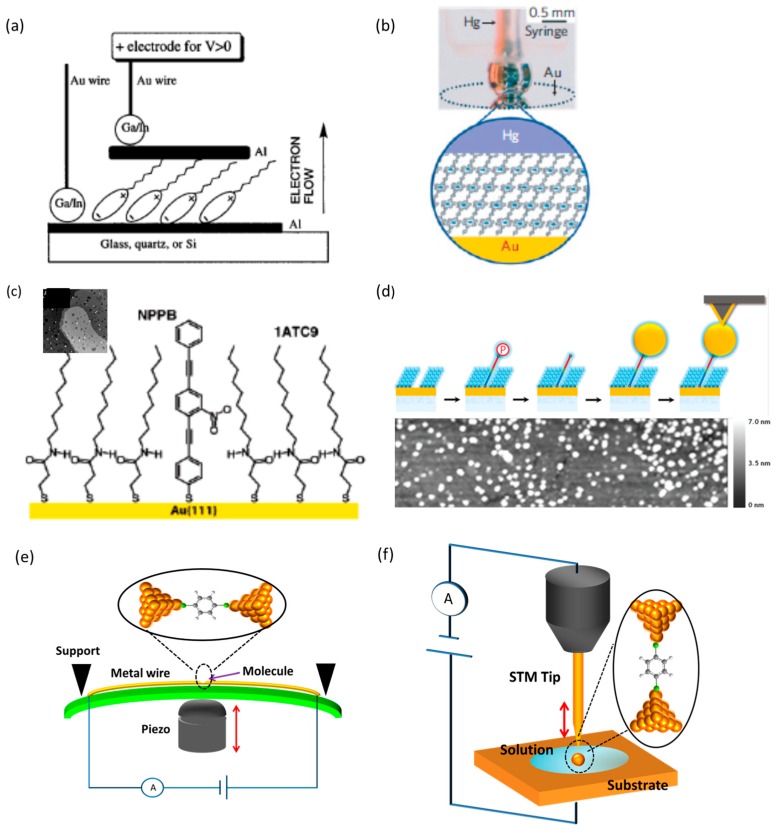
(**a,b**) Schematic illustration of the molecular junctions with small number of molecules. (**a**) The metal pad is deposited on the film. The Au wire is connected with the metal pad by eutectic Ga/In [12]; (**b**) Photograph and schematic representation of the Au/monolayer film/Hg junction. The hanging Hg drop electrode is used as the top electrode [21]; (**c**–**f**) Schematic illustration of the single molecule junctions; (**c**) The target molecule is inserted into the host alkanethiol-based-SAM matrix. Inset STM image: (140 nm × 140 nm, sample bias = −1.0 V, and tunneling current = 2 pA [22]; (**d**) (Top) sample preparation for Au nano particle/molecule/Au junction. Au nano particle is covalently bond to target molecule. A Au coated AFM cantilever contacts the Au nano particle. (Bottom) AFM image of the sample [23]; (**e**) Schematic view of MCBJ technique; (**f**) Schematic view of STM-BJ technique.

To investigate the rectification properties of molecules, electronic contact between molecules and external metal electrodes must be established. In the following, we will explain the fabrication and electronic characterization techniques of molecular junctions consisting of molecular assembly. In a pioneering work on the molecular diode, the LB technique was used to control molecular orientation in a molecule film and make electronic contact between the molecular film and a metal electrode [12]. In subsequent research, the typical fabrication approach was based on the self-assembly of molecules on a metal surface. In the first step, SAM film of a target molecule was formed on a metal electrode by immersing a metal electrode in a solution containing the target molecules. In the self-assembly process, molecular orientation with respect to the metal electrode can be controlled. Usually, to make electronic contact between a molecule and metal electrodes, anchoring groups with large chemical affinity to metal electrodes are introduced at the two termini of a molecule [17,18,19,20]. Suppose that a molecule has two kinds of anchoring groups with weak and large affinities to a metal electrode, the anchoring group with the larger affinity to the metal electrode preferentially binds to the metal electrode in the self-assembly process. Consequently, molecular orientation with respect to the metal electrodes can be controlled in the molecular film. This approach was successfully demonstrated to control a molecular orientation where the chemical affinity was controlled by protecting and deprotecting a chemical functional group at a terminal of a molecule [9]. After the first step of making the electronic contact between one terminus of a molecule and a metal electrode (i.e., bottom electrode), another electrode (i.e., top electrode) was placed in contact with the molecular film to prepare molecular junction-structure. Figure 2a,b shows a schematic illustration of the molecular junctions [12]. The top electrode can be deposited on top of the SAM film (Figure 2a). The direct deposition of metals on the SAM films by using electron-beam or thermal evaporation can cause atomic-level contact between top and bottom electrodes, and also damage the organic monolayer by reaction with hot metal vapors. To avoid short circuits of the junctions and the damage to the molecules, liquid metals (e.g., eutectic GaIn and Hg) can be also used for the top electrodes [21] (Figure 2b). To fabricate a molecular junction on the single molecule scale, matrix isolation approach of individual molecules has been developed [22] (Figure 2c,d). In this approach, an insulating host alkanethiolate-based SAM matrix was prepared on a metal substrate, and the substrate was immersed into the solution containing a conductive target molecule. The target molecule can absorb on defect sites of the host matrix and/or replace host molecules in the SAM matrix. Figure 2c shows a scanning tunneling microscopy (STM) image of individual NPBB (4-(2′-nitro-4′-phenylethynyl-phenylethynyl)-benzenethiol) molecules isolated in an insulating host alkanethiol-based SAM matrix, in which individual conductive NPBB molecules are imaged as bright spots (Figure 2c) [22]. Based on the matrix isolation approach, a single molecular junction-structure was prepared by connecting a top electrode of a Au nanoparticle onto the end of the molecule (Figure 2d) *I*-*V* measurement of the single-molecule junction is performed by positioning a AFM tip atop the Au nanoparticle [23].

After the matrix isolation approach, an alternative approach to preparing single-molecule junctions was developed on the basis of the break junction technique. Mechanically controllable break junction (MCBJ) and STM-based break junction (STM-BJ) are the most widely used techniques to fabricate the single-molecule junctions [14,15,16]. In the MCBJ technique, notched metal wires are fixed onto a flexible substrate (Figure 2e). The substrate is fixed to a support, using a three-point bending configuration. By mechanically bending the substrate using a piezoelectrically controlled push-rod, a single atomic contact of a metal is formed just prior to breaking the metal wire. After the wire is broken, a nano gap is formed. By inducing guest molecules into the nano gap from solution or gas phases, a guest molecule bridges the nano gap and forms a single-molecule junction. In the STM-BJ technique, molecular junctions are formed between a nano gap made by breaking a metal point contact between a STM tip and a metal substrate (Figure 2f).

## 3. Mechanism of the Rectification Property of the Molecular Diode

Here, we explain two mechanisms of the rectification properties of the molecule diode. Beside the Schottky barrier formation at the metal-molecule interface in the molecular junction, there were two main origins of the rectification properties of the molecular junctions. The first one is based on sequential tunneling though acceptor (A) and donor (D) types of molecular units in the molecular junction. The rectified charge transport though the acceptor and donor units mimics that of the p-n junction of the semiconductor rectifier. The second one is based on asymmetric electronic coupling at the two metal-molecule interfaces in the molecular junction.

**Figure 3:**
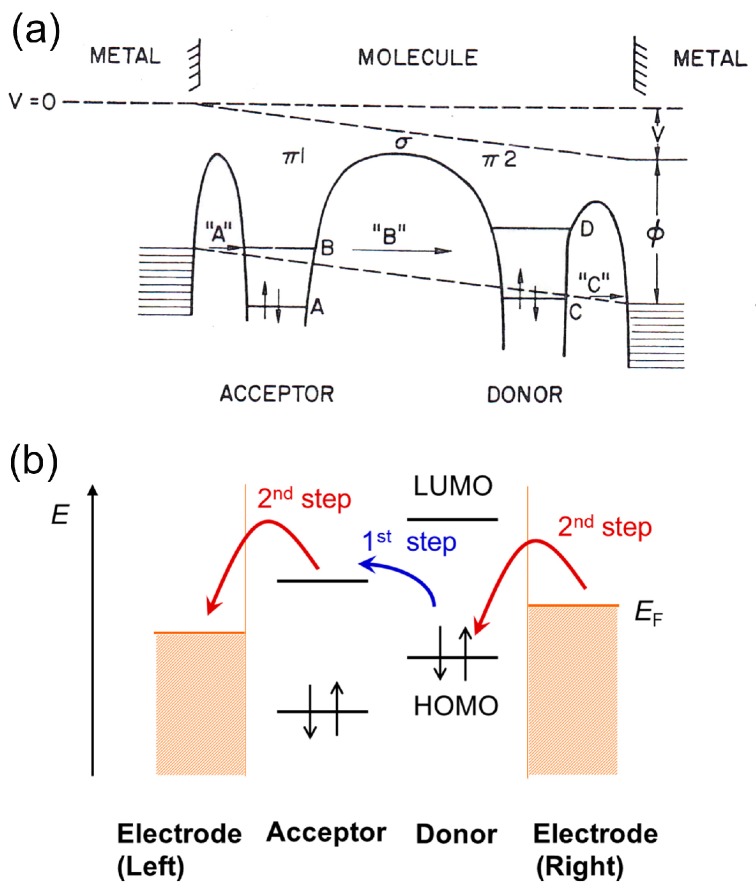
(**a**) Energy level diagram of the molecular diode proposed by Aviram and Ratner [11]. B and D are LUMO, and A and C are the HOMO levels of acceptor and donor, respectively. The positive bias voltage is applied to the molecular diode, and energy level shifts. “A”, “B” and “C” are tunneling process. Electrons preferentially flow from acceptor to donor; (**b**) another possible charge transfer process, in which electrons preferentially flowed from donor to acceptor (For a detail, see main text).

The first mechanism of the sequential tunneling is proposed by Aviram and Ratner [11]. In the model (Figure 1a), the LUMO (lowest unoccupied molecular orbital) of the acceptor unit and the HOMO (highest occupied molecular orbital) of the donor unit is close to the Fermi level of the electrodes. Figure 3 shows the energy diagram of the molecular diode under forward bias condition. When the left electrode is negatively biased relative to the right electrode, electron transfer onto the acceptor becomes possible as soon as the applied field becomes large enough for the Fermi level of the left electrode to overlap the acceptor level. A similar process occurs at the donor end, where electron transfer from the donor to the right electrode becomes possible when the applied voltage is *V* > *IP − W* where *IP* is donor ionization potential and *W* is the work function of the metal electrode. Motion of electrons from acceptor to donor occurs under the action of the field via tunneling process. When polarity is reversed, donor level should be lowered to the Fermi level of the right electrode and the Fermi level of the left electrode should be lowered below the acceptor level in order to obtain the tunneling through these levels. Therefore, the threshold voltage for this process is high, which explains rectification properties of the molecular diode. In this model, electrons preferentially flowed from A (acceptor) to D (donor) as a result of the two step charge transfer process. The first step is an electron moving from the left electrode to the LUMO of the acceptor unit and an electron moving from the HOMO of the donor unit to the right electrode (see Figure 3a) and the second step is an internal relaxation of the resultant zwitterion (i.e., A^−^–D^+^) to the ground-state. There is another model of the sequential tunneling though the acceptor and donor types of the molecular units. In this model, the first step of the charge transfer process is an electron moving from the HOMO of the donor unit to the LUMO of the acceptor unit (i.e., formation of A^−^–D^+^). This is followed by an electron moving from LUMO of the acceptor unit to the left electrode and an electron moving from the right electrode to the HOMO of the donor unit (see Figure 3b). The overall direction of preferred electron flow is from D (donor) to A (acceptor) [5] and is opposite to that of the Aviram and Ratner model (i.e., anti-Aviram and Ratner model).

**Figure 4:**
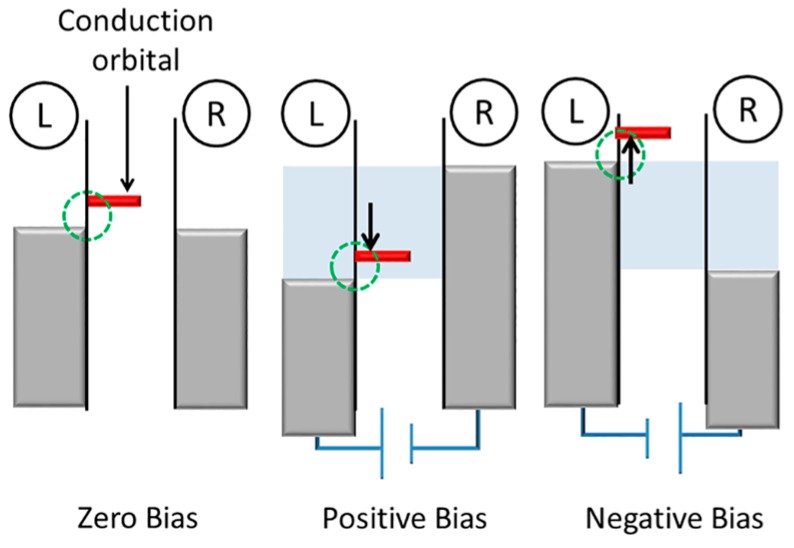
Energy diagram of the molecular diode. Here, the conduction orbital is LUMO, and the electronic coupling between LUMO and left electrode is larger than the right electrode. When the left electrode is positively biased relative to the right electrode, LUMO is within the bias window, which generates large current. Blue region corresponds to the bias windows at negative and positive bias conditions.

Another mechanism originates from an asymmetric molecule-metal coupling in the molecular junction. Here we assume that the conduction orbital of the molecular junction is LUMO and the LUMO effectively hybridizes with the left electrode (Figure 4). Owing to the asymmetric molecule-metal coupling, the conduction orbital mainly follows the Fermi level of the left electrode. When the left electrode is positively biased relative to the right electrode, the LUMO is shifted within the bias window. An electron can be transported via this molecular orbital [24], leading to large current. On the other hand, at the opposite bias voltage, the LUMO is pushed away from the bias window, resulting in a rectified current at positive bias.

## 4. Molecular Diode with Small Number of Molecules

**Figure 5:**
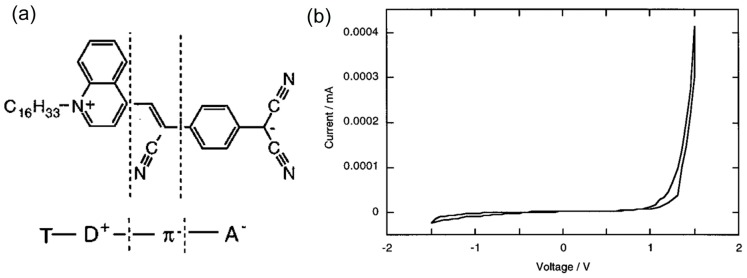
(**a**) Chemical structure of γ-(*n*-hexadecyl)quinolinium tricyanoquinodimethanide (C_16_H_33_Q-3CNQ). T is the hexadecyl tail, D^+^ is the quinolinium moiety, π is the π-electron bridge, and A^−^ is the tricyanoquinodimethanide (3CNQ-) moiety; (**b**) Current-voltage characteristic of single monolayer of C_16_H_33_Q-3CNQ sandwiched between Al electrodes using eutectic Ga/In and Au wires [12].

In 1997, Metzger et al. confirmed the first molecular diode with monolayers of asymmetric γ-(*n*-hexadecyl)quinolinium tricyanoquinodimethanide, C_16_H_33_Q-3CNQ [12] (Figure 5a). The C_16_H_33_Q-3CNQ can be regarded as a T-D^+^-π-A^−^ molecule, where T is the hexadecyl tail, D^+^ is the quinolinium moiety, π is the π-electron bridge, and A^−^ is the tricyanoquinodimethanide (3CNQ^−^) moiety [25]. The LB films of C_16_H_33_Q-3CNQ monolayer film was transferred to a base Al electrode, and an Al electrode was deposited on top of the LB film. Each electrode was connected to a Au wire by a eutectic Ga/In. The molecular orientation in the film was controlled using the LB technique: the hydrophilic 3CNQ^−^(A^−^) end was placed closest to the base Al electrode. The base electrode was grounded, and the bias voltage was applied to the top electrode. Figure 5b shows the *I–V* curve of the molecular junction of C_16_H_33_Q-3CNQ. Larger current flowed at the positive bias voltage above 1.0 V, indicating that the electrons preferentially flowed in one direction from 3CNQ(A^−^) to quinolinium (D^+^) (i.e., Electrons preferentially flowed from A (acceptor) to D (donor) in the T-D^+^-π-A^−^ molecule (see Figure 3a)). This pioneering study put forward several issues including reproducibility of the device fabrication and the device performance of the molecular diode study. Of 39 devices, 17 were electrical short circuits, either because of monolayer defects or because the eutectic Ga/In made defects. Among 22 good devices, four exhibited rectifying behavior with rectification ratio of 2.4~26.4. The threshold voltage varied from junction to junction in the range *V* = 0.8–1.3 V. As the cycle of the *I–V* measurement was repeated, the rectification ratio dropped steadily and disappeared after 4–6 cycles. It appeared that, under the intense electric fields, the molecular dipoles reoriented to minimize energy. Since the pioneering work of the molecular diode, intensive research effort has been focused on the diode properties of the T-D^+^-π-A^−^ type molecules and related compounds and it is confirmed that the T-D^+^-π-A^−^ type molecules overwhelmingly favors the electron transport from D (donor) to A (acceptor) [5] (see Figure 3b).

**Figure 6:**
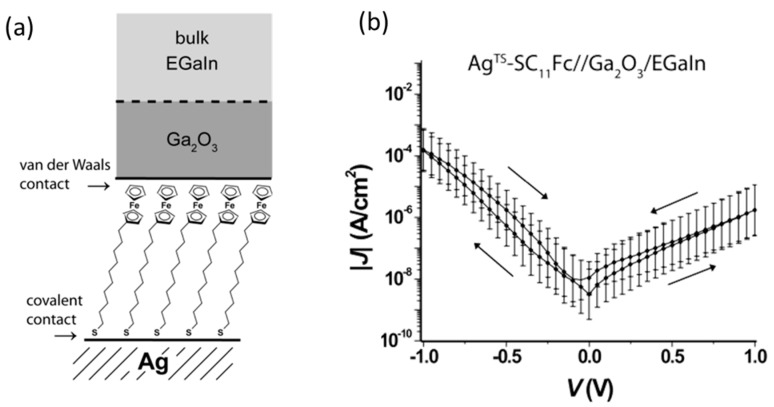
(**a**) Schematic representation of the tunneling junction with the structure Ag/alkanethiolates with ferrocene (Fc) head groups (SC_11_Fc)//Ga_2_O_3_/eutectic Ga/In; (**b**) Average traces of the absolute value of the current density, |*J*|, plotted versus applied voltage for all Ag/SC_11_Fc//Ga_2_O_3_/eutectic Ga/In junctions [26].

To improve the low yield and reversal of the rectification properties of molecular junctions, a SAM-based fabrication approach has been developed. As mentioned above, molecular orientation was controlled in the molecular self-assembly process on a metal electrode and molecules strongly bind to the bottom electrode using anchoring groups at the molecular termini. To make good and stable electronic contact between molecules in the SAM and a top electrode, liquid metals have been utilized. Nijhuis et al. used an eutectic alloy of gallium and indium (eutectic Ga/In) as a top electrode of molecular junctions [26] (Figure 6a). A eutectic Ga/In is a liquid at room temperature, but its spontaneously formed surface oxide (Ga_2_O_3_) skin gives it apparent non-Newtonian properties and allows it to be molded into conically shaped tips. These tips formed soft electrical contacts with SAM of alkanethiolates with ferrocene head groups (S(CH_2_)_11_Fc), and formed stable tunneling junctions in high (70–90%) yields. The bottom electrode was grounded, and the bias voltage was applied to the top electrode. *I–V* measurements showed that the large current flowed at the negative bias voltage (Figure 6b). The rectification ratio was 100. The theoretical calculation revealed that the transmission was dominated by HOMO, and the HOMO lay principally on the ferrocene unit. The HOMO level followed the Fermi level of the top electrode attached to the ferrocene. Negative bias drove the HOMO towards the bias window, and the positive bias pushed it way from the bias windows, resulting in a larger current at the negative bias voltage.

Similar measurements have been performed for the 2,2′-bipyridyl-terminated *n*-alkanethiolates (S(CH_2_)_11_-4-methyl-2,2′-bipyridyl) [27]. The bipyridyl unit is coupled to the top electrode of Ga_2_O_3_/eutectic Ga/In. The bottom Ag electrode was grounded, and the bias voltage was applied to the top electrode. A larger current flowed at positive voltage, which was opposite to that of the ferrocene SAM. The theoretical calculation revealed that the transmission was dominated by LUMO, which was different from S(CH_2_)_11_Fc, and explained the opposite rectification direction. Significant rectification ratios starting at *V* = 0.5 V and reached to 85 at 1.0 V.

To improve the rectification ratio of the molecular diode, detailed studies on the structural and electronic relationship of the molecular junctions have been performed. Yuan et al. investigated the relationship between surface topography of the bottom Ag electrodes and rectification properties of the junctions based on the ferrocene SAM of S(CH_2_)_11_Fc [28]. They used a liquid metal alloy eutectic Ga-In as the top electrode, and prepared the bottom electrode with the two different methods: (i) combination of annealing and template-stripping (Ag^A−TS^); and (ii) direct deposition (Ag^DE^) on Si/SiO_2_. Junctions with SAMS on Ag^A−TS^ surface had a high rectification ratio, ~100, while junctions with Ag^DE^ surfaces had a poor rectification ratio of 10. AFM was used to analyze the topography of the bottom electrode. The root-mean-square roughness was 0.82 nm for Ag^A−TS^ and 5.1 for Ag^DE^. They concluded that the number of defects increased for the rough surface, and at defects sites the SAMs were disordered and the Fc units were randomly oriented and therefore could not block the current in the off state, resulting in small rectification ratios. By studying the various surfaces, they also showed that the root-mean-square (rms) surface roughness was not the only crucial factor, but peak-to-valley roughness, number of grains, and width of the grooves between the grains were all important to obtain molecular junctions with high rectification ratio.

**Figure 7:**
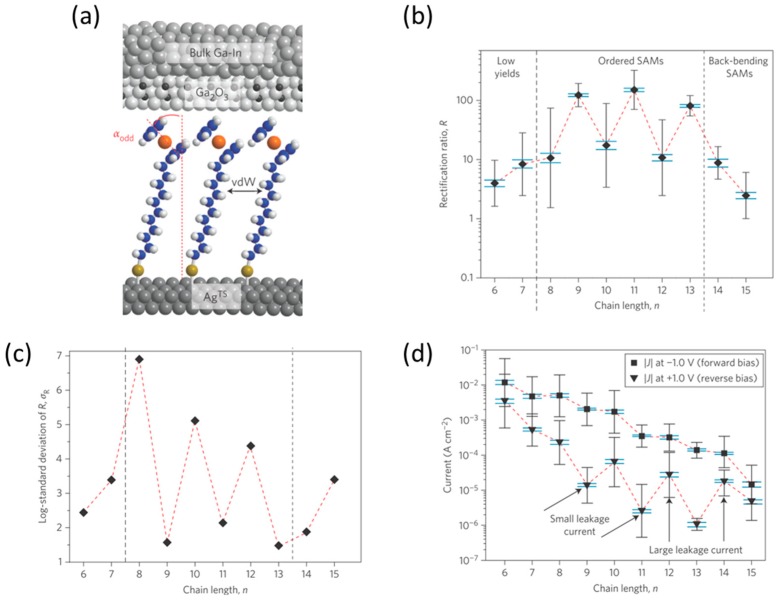
(**a**) Schematic illustration of junctions of Ag-SC*_n_*Fc//Ga_2_O_3_/eutectic Ga/In; (**b**) Rectification ratio as a function of alkyl unit *n*; (**c**) Value of tilt angle as a function of *n*, derived from NEXAFS spectra; (**d**) Values of current measured at +1.0 and −1.0 V, as a function of *n* [29].

The relationship between quality of the molecular film and related rectification properties has been investigated for the ferrocene-alkanethiolate SAM of S(CH_2_)*_n_*Fc (*n* = 6, 15) using a liquid metal alloy eutectic GaIn as the top electrode [29]. The substrate was grounded, and bias voltage was applied to the top electrode. A larger current flowed at the negative voltage. Figure 7b shows the rectification ratio as a function of alkyl unit *n*. The junctions with *n* = 9, 11 and 13 displayed large rectification ratio of the order of 10. Odd-even effect was verified in a ferrocene alkanethiolate SAM. The NEXAFS measurements showed that the tilt angle of the Fc units with respect to the surface normal (shown in the Figure 7c) was on average ~5° smaller (that is, the Fc units were standing up more) for SAMs with *n*_odd_ on the substrates than for SAMS with *n*_even_. The more upright Fc units in molecular diodes consisting of SAMs on the substrate with *n*_odd_ packed better and were stiffer because of more favorable molecule-molecule interactions. These SAMs were more stable during fabrication, resulting in working devices with high yields that blocked the current efficiently at reverse bias (Figure 7d), resulting in large rectification ratios.

**Figure 8:**
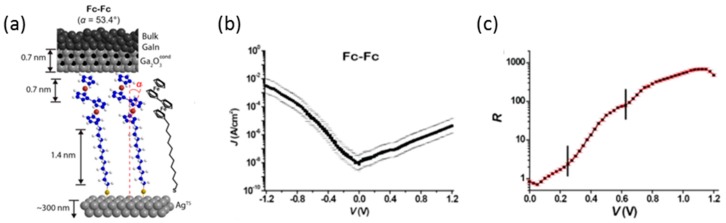
(**a**) Schematic illustrations of the junctions of Ag/SC_11_Fc_2_//Ga_2_O_3_/eutectic Ga/In; (**b**) Log-average *J*(V) plot of the junctions with SAMs of SC_11_Fc_2_; (**c**) The rectification ratio versus applied bias [13].

The rectification ratio can be improved by increasing the number of the conduction orbitals. Yuan et al. investigated the molecular diode of biferrocene using a liquid metal alloy eutectic Ga-In as the top electrode [13]. The substrate was grounded, and bias voltage was applied to the top electrode. Figure 8 shows the *I–V* curve for the monolayer film of SC_11_Fc_2_ (Fc = ferrocenyl) on Ag substrate. A larger current flowed at the negative bias voltage. The rectification ratio increased sharply above 0.2 V. Beyond 0.6 V, another sharp increase of rectification ratio was visible, and the rectification ratio reached to 1000 at 1.0 V. The two-step increase was explained as follows. Both HOMO and HOMO-1 are close in energy to the Fermi levels. Both orbitals lay principally on the biferrocene unit, and they were coupled to the top electrode. At relatively low negative bias, the HOMO level fell within the bias window at relatively low negative bias. By increasing the bias voltage, the HOMO-1 came into the bias window providing a second tunneling channel, that is, through HOMO and HOMO-1. Consequently, this biferrocene diode with two conduction orbitals had much higher currents in the on-state than diodes with only one conduction orbital.

## 5. Single Molecular Diode

**Figure 9:**
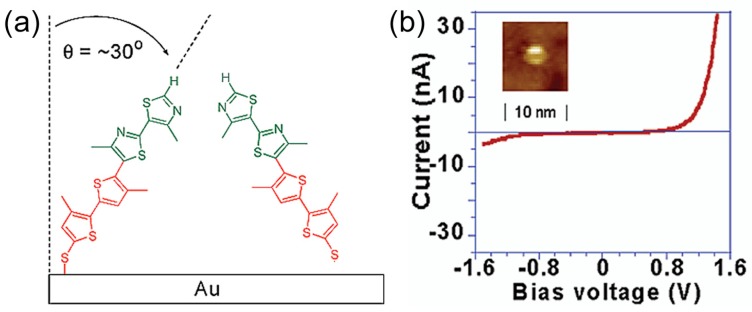
(**a**) Schematic drawing of conjugated diblock molecules on Au substrate; (**b**) *I–V* curve of isolated molecules of diblock molecule inserted into pre-assembled monolayers of 1-decanethiol on Au/mica (setpoint conditions: +1000 mV and 2 pA). (Inset) Constant-current STM image [30].

The rectification properties have been investigated for molecular junctions with the assembly of molecules. Rectification property of a single molecule junction was demonstrated by using the matrix isolation of individual active molecules, diluted in a sea of inactive ones. Ng et al. investigated the single molecular diode based on a conjugated diblock co-oligomer [30] (Figure 9a). The diblock molecule consisted of an electron-rich bithiophene (D) segment and an electron-poor bithiazole (A) segment. A disulfide was introduced at the thiophene end, so that the diblock molecule was adsorbed on a Au surface, where the diblock molecule preferentially oriented itself with the bithiophene bound to the substrate. The diblock oligomer was inserted into a SAM of the alkanethiolate host. Individual diblock molecules were widely separated. The bright spot in the inset of Figure 9b represents the STM image of diblock molecule inserted into preassembled monolayers of decanethiol. The bright spots all over the monolayers of decanethiol were very uniform in size. Figure 9b shows the *I–V* curve of the single diblock molecule measured with STM. Here, the tip was grounded. The larger current flowed at the positive bias voltage, which means electrons preferentially flowed from A (acceptor) to D (donor) (see Figure 3a). These results agreed with the macroscopic p–n (A–D) junction and Aviram-Ratner model.

**Figure 10:**
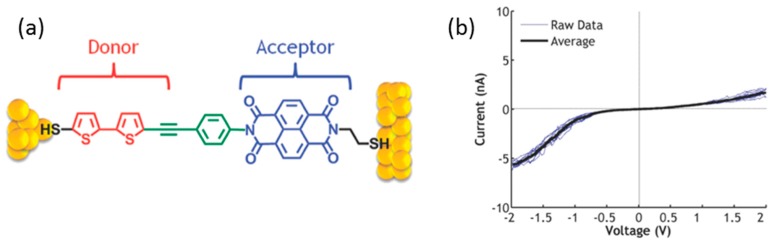
(**a**) Structure of the bithiophene-phenylacetylene-naphthalenediimide-dithiol (BPNDT) molecular junction; (**b**) *I–V* curve of a BPNDT molecular junction showing the average *I–V* curve over 10 traces [23].

Yee et al. utilized the Au nano particle as the top electrode for the single molecular junction [23]. The bithiophene-phenylacetylene-naphthalenediimide-dithiol (BPNDT) molecule was inserted into the decanethiolate SAM initially created on a smooth Au surface, which caused an individual BPNDT molecule to be widely separated (Figure 10a). The BPNDT molecule has a trimethylsilyl (TMS) group protecting the thiol closest to the donor. Since only one thiol-binding group was exposed, the molecule preferentially oriented itself with the acceptor bound to the substrate. Upon bonding to the substrate, the protecting TMS group was removed. The 5 nm Au nano particle was covalently bound to the unprotected end of BPNDT molecule. The electric measurement was performed with conductive AFM. The tip was biased relative to the substrate. Figure 10b shows the *I–V* curves of the single BPNDT molecular junction. Larger current were seen to be present under negative bias. Electrons preferentially flowed from D (donor) to A (acceptor) (see Figure 3b). This behavior was opposite to that of a macroscopic p–n (A–D) junction. The theoretical calculation revealed that the transmission was dominated by the HOMO, and HOMO lay principally on the donor (i.e., bithiophene) with negligible weight on acceptor (i.e., naphthalenediimide), and strongly hybridized with the thiol end group. The HOMO level followed the Fermi level of the Au electrode attached to the donor. The negative bias drove the HOMO towards the bias window, and the positive bias pushed it away from the bias windows, resulting in a larger current for negative bias.

**Figure 11:**
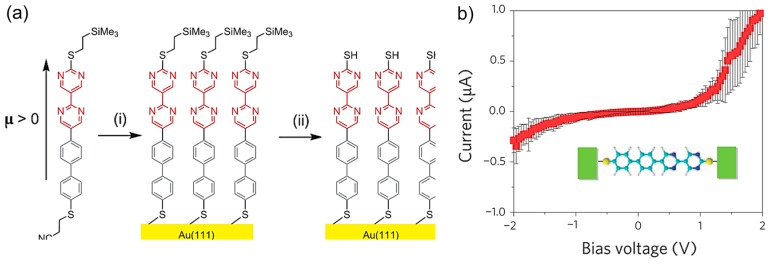
(**a**) Preparation of the SAM of non-symmetric dipyrimidinyl–diphenyl molecule. The reagents used were (i) sodium ethoxide, ethanol and THF, and (ii) tetrabutylammonium fluoride and THF; (**b**) Average curves for the single-molecule junctions of the non-symmetric dipyrimidinyl–diphenyl built from 50 individual *I–V* curves [9].

The pioneering work of the single molecular diode based on the BJ technique was reported by Tao’s group in 2009 [9]. They fabricated the diblock molecular diode, where an electron deficient bipyrimidinyl (A) moiety covalently connected to an electron-rich biphenyl (D) block. The diblock molecule resembles the Aviram-Ratner model molecule. The asymmetric diblock molecule was terminated with two different protecting groups, trimethylsilylethyl (dipyrimidinyl side) and cyanoethyl (diphenyl side) (Figure 11a). They controlled the molecular orientation as the following process. The first de-protection step removed the cyanoethyl protecting group, which allowed a SAM to form on the gold substrate of the diblock non-symmetric molecules with the diphenyl end bound to the substrate electrode. The second step removed the trimethylsilylethyl group, which exposed the thiol group at the dipyrimidinyl end to the tip electrode (Figure 11a). The single molecular junction was fabricated by STM-BJ technique. The tip was grounded, and the bias voltage was applied to the substrate. The resulting asymmetric single-molecule junction exhibited pronounced rectification behavior, with current flowing from the dipyrimidinyl to the diphenyl moieties (Figure 11b). Electrons preferentially flowed from A (acceptor) to D (donor) (see Figure 3a). The average rectification ratio at a 1.5 V bias was about five to one from positive to negative bias polarities. To ensure that the orientation of the molecules did not change during the measurement, they analyzed the polarities of the individual I–V curves and found that more than 90% of the curves had the same rectification polarity, which indicated that the orientation remained unchanged. Currently, there are several reports of single molecular diode with various molecules including diblockmolecule, DNA, π-stacked molecule, transition metal complexes [31,32], hydroxyphenylpyridine unit [33], NiPc/PB [34], ABT [35], α,ω-dithiols [36], DPE-2F [37], diphenyl-oligoene backbones [38], two phenyl-ethynyl–phenyl-systems [39], polyoxometalate [40], H_2_-TPP and Co-TPP [41], pyridinoparacyclophane-based diodes consisting of cyclophane moiety as the bridging group between the p-type biphenyl unit and the n-type bipyrimidinyl unit [42].

**Figure 12:**
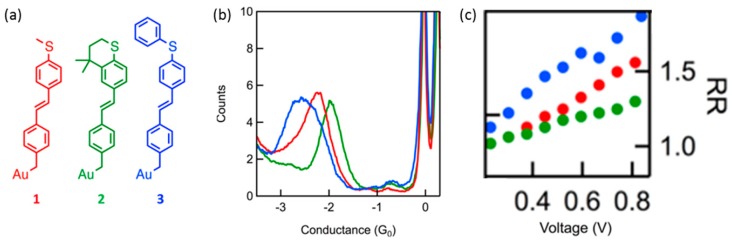
(**a**) Chemical structures for the molecular rectifier design (molecule (**1**)), along with two modified designs, (**2**) and (**3**): The stilbene molecular backbone with a single methylsulfide linker at the 4 position and a covalent gold-carbon bond at the 4′ position (molecule 1); (**b**) Log-binned conductance histograms for the three molecules; (**c**) Rectification ratio as a function of bias voltage [42].

While the initial single-molecule study focused on the rectification that associated with the D^+^-A^−^ type molecules, Batra et al. studied the rectification from asymmetric metal-molecule electronic coupling in a single-molecule junction [42]. They investigated the single molecular diode with a stilbene molecular backbone. The symmetric stilbene backbone bound to Au electrode via a covalent Au-C bond at one of the interface. At the other side of the interface, the backbone bound to Au electrode via Au-S bond, and the strength of the π-Au-S coupling was controlled by using the three anchoring groups, as shown in Figure 12a. The coupling was better for (**2**), and worse (**3**) than (**1**) based on their chemical structure. The methylsulfide group in (**2**) was locked in-plane with the molecular backbone through the saturated six membered ring. The zero bias conductance showed that molecules (**2**) and (**3**) conducted better and worse than molecule (**1**) and confirmed that the coupling between the backbone and the Au-S bond was tuned through chemical modifications. For all three molecules, rectification increased linearly with applied bias, with significant asymmetry seen as low as 0.5 V. The molecule with the poorest coupling, (**3**), rectified the most, while (**2**), with the strongest coupling, rectified the least. They discussed that the strong Au-C bond resulted in a hybrid Au-molecule gateway state pinned close to the Fermi level of one electrode. The energy of this state shifted with applied bias, resulting in rectification at rather low bias voltage.

**Figure 13:**
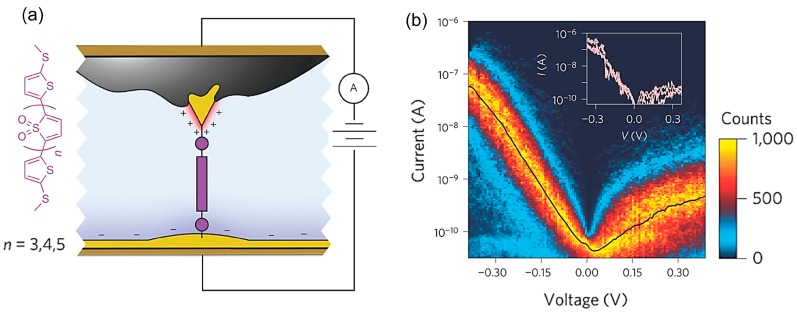
(**a**) Molecular structure of oligomer consisting of thiophene-1,1-dioxide units flanked by two methyl-sulphide-bearing thiophenes (TDO*_n_*), and schematic of the molecular junction created using asymmetric area electrodes; (**b**) Two-dimensional absolute current versus voltage histogram for TDO_5_ in PC. Inset: Examples of exceptionally rectifying junctions (three selected traces) [43].

Capozzi et al. improved the rectification ratio of the single-molecule diode through environmental control [43]. Figure 13b shows the *I–V* response of the single oligomer consisting of four thiophene-1,1-dioxide units flanked by two methyl-sulphide-bearing thiophenes (TDO4) in polar aprotic solvent, propylene carbonate (PC) (Figure 13a). The bias voltage was applied to the tip relative to the substrate. The *I–V* response illustrated a clear asymmetry in current with bias sign, showing a much higher current at negative voltage than at positive voltage. The rectification ratio was greater than 200 at voltage as low as 370 mV. It should be noticed that the TDO4 is a symmetric molecule. The current rectification was observed for this symmetric molecule. The diode property was not observed in a nonpolar and non-ionic solvent, but was observed only in polar aprotic solvent. The current rectification was observed for other molecules that did not belong to the TDO family: 4,4′-bipyridine (molecule (**1**)), 4,4 diamino-p-terphenyl (molecule (**2**)). Molecules of (**1**) and (**2**) displayed “on” behavior at negative and positive bias, respectively. The opposite bias polarity was explained by the difference in the conduction orbital. The previously reported theoretical study showed that molecule (**1**) conducted through the LUMO, whereas molecule (**2**) conducted through the HOMO. The observed current rectification in polar aprotic solvent was explained by the asymmetric bias dependent electric double layer. In polar aprotic solvent, ions moved to screen out the electric field due to charges on the metal, forming the electric double layer: therefore this is an environment-induced effect, and not really a single-molecule effect. This electric double layer influenced the electrostatic environment around the junction. The asymmetry in the electrode areas exposed to the solvent resulted in the formation of a denser double later in the tip electrode when compared with the substrate. This resulted in the pinning of the molecular orbital to the chemical potential of the substrate, yielding a current dependence on the polarity of the applied bias.

**Figure 14:**
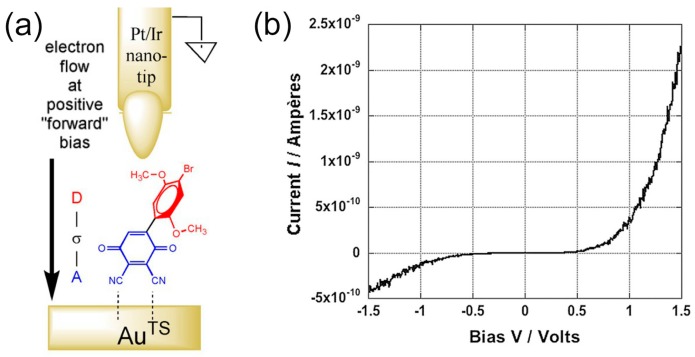
(**a**) Self-assembled monolayer (SAM) of hemibiquinone on template-stripped Au surface (Au^TS^) interrogated by an STM Pt/Ir tip atop the SAM. An electron-rich donor (D) electronically and spatially separated by an insulating bridge (σ) from an electron-poor acceptor (A) [44]. The electrical ground is shown; (**b**) Averaged 50 *I–V*s of the molecular junction in (**a**).

Metzger et al. reported robust and high rectification ratio of a very small molecular rectifier using an A-σ-D type molecule of hemibiquinone (HBQ) (Figure 14a) [44]. A single C-C biphenyl bond in the HBQ molecule has an appreciable twist angle (ca. 40°), which prevents full π-orbital conjugation and provides the necessary molecular orbital isolation for rectification [11]. They showed that for rectification, insulating bridge (σ) can be as short as a single bond. The HBQ molecule was targeted because of its potential to self-assemble through interaction of the nitrile groups with Au surface (see the schematic illustration in Figure 14a). The reification of HBQ was investigated by measuring scanning tunneling spectroscopy (STS) atop a SAM of HBQ on Au^TS^ using an STM Pt/Ir tip. Figure 14b shows the STS spectra as the average of over 50 repetitive STS spectrum, nominally over the same spot in the sample. When there is no molecule under the STM tip, the current is symmetrical about ±V. When the molecule is under the STM tip, the positive bias current for a single molecule leads to rectification ratio (RR) = 3 at 1 V, and RR = 6 at 1.5 V and electrons preferentially flowed from D (donor) A (acceptor) (see Figure 3b).

**Figure 15:**
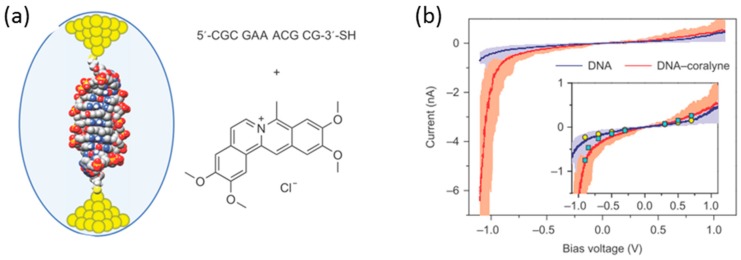
(**a**) Molecular junction composed of the DNA(5′-CGCGAAACGCG-3′-SH)–coralyne (right) complex; (**b**) Average *I–V* curves (solid line) over 40 individual curves (light shadow) of native DNA (blue) and DNA–coralyne complex (red). Inset: Graph overlay of *I–V* curves with static current values (yellow circles, native DNA; cyan squares, DNA–coralyne complex) under different bias voltages [45].

Along with the studies on the single-molecule diodes of the small organic molecules, single-molecule diodes based on biological large molecules such as DNA have been investigated owing to the inherent structural and molecular recognition properties that could be ideal for molecular electronics applications. Guo et al. fabricated a DNA-based rectifier with high rectification property using STM-BJ [45] (Figure 15a). The DNA-coralyne complex was prepared by intercalating two coralyne molecules in a 11 base pair DNA molecule (5′-CGCGAAACGCG-3′-S) containing three mismatched A-A base pairs at the center. Coralyne is a small, planar molecule that strongly binds with adenine-adenine (A-A) base pair mismatches. Figure 15b shows the *I–V* response of single native DNA and DNA-coralyne complex, where the tip was grounded, and the bias voltage was applied to the substrate. The native DNA single molecular junction did not show rectification behavior, while the DNA-coralyne single molecular junction showed rectification with a high rectification ratio around 15 at 1.1 V. This rectification ratio was close to the theoretically estimated upper limit of 20 that could be achieved in a coherent transport molecular junction system. The theoretical study revealed that the rectification behavior was caused by the coralyne induced local spatial asymmetry of the distribution of electron states along the DNA chain. The wavefunction weights at the left edge of the molecule were 10,000 times larger than that at the right edge. This asymmetry of the distribution of electron states led to a change in the coupling between the HOMO-1 orbital and the electrode when an external voltage was applied.

**Figure 16:**
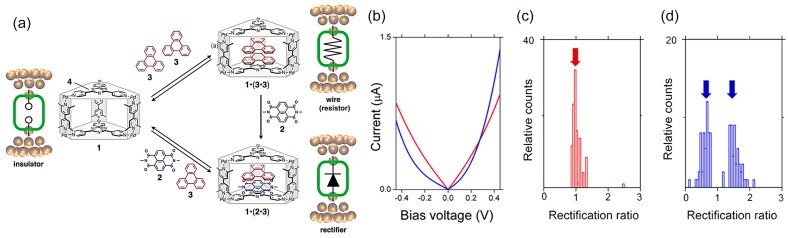
(**a**) Schematic representation of single-molecule junctions sandwiched by two Au electrodes and corresponding electronic components of junctions; (**b**) *I–V* curves without and with rectification properties for the homo (red) and hetero (blue) complexes with electronic conductance of 10^−3^–10^−2^ G_0_ at a bias voltage of 0.1 V; (**c**,**d**) Histograms of the rectification ratio at a bias range of 0.4–0.5 V for the (**d**) homo and (d) hetero π-stacked complexes. The mean conductance values in the bias voltage range of +0.4 (−0.5) to +0.5 (−0.4) V were calculated for a positive (negative) polarity. The rectification ratio was calculated as R = mean I_+_/mean I_–_, where R is the rectification ratio, and mean I_+_ and mean I_–_ are the mean absolute current values in the negative and positive bias regions, respectively. The arrows indicate sharp peaks with a rectification factor of one for the homocomplex (blue). In addition to a peak with a rectification factor of one, broad distributions with rectification factors of approximately 0.73 and 1.38 are indicated by arrows for the heterocomplex (red) [46].

The single-molecule diode with a host–guest system has been demonstrated using a self-assembled molecular cage containing aromatic stacks [46]. In typical single-molecule diodes, the molecules in the junction were connected by chemical bonds, and therefore the entire electronic functionality was unchanged. The host-guest system adds tunability of chemistry and related electronic functions to the molecular junctions (Figure 16a). The molecular cage can accommodate a naphthalenediimide **2** and triphenylene **3** pair or a dimer of **3**, in which the enclosed aromatic pair is bookended by the electron-poor triazine panels **4** of the cage. The empty cage **1**, homo π-stacked complex **1**·**(3·3)**, and hetero π-stacked complex **1·(2·3)** behave like an insulator, a resistor, and a rectifier, respectively. STM-BJ measurements revealed that both the homo and hetero π-stacked complexes **1·(3·3)** of **1·(2·3)** indicated conductive characteristics with electronic conductance values of 10^−3^–10^−2^ G_0_, while the empty cage exhibited an insulating characteristic with ~10^−5^ G_0_. STM-BJ-based *I–V* measurements were performed to confirm the electronic functionality of the π-stacked complexes, for which an additional diode property with a rectification ratio of 1.4–2.0 was determined for the hetero π-stacked complex **1·(2·3)**, as shown in Figure 16b–d. Theoretical calculations demonstrate that this rectification behavior originates from the distinct stacking order of the internal aromatic components (i.e., **1·(2·3)** and **1·(3·2)**) against the electron-transport directions and the corresponding lowest unoccupied molecular orbital conduction channels localized on one side of the molecule **2** in the molecular junction.

## 6. Conclusions

In this review, we described the technique that measures the electron transport properties of molecular junctions with a small number of molecules and a single molecule. On the basis of LB and self-assembly methods in combination with the liquid metal electrode, the molecular orientation controlled diode with a small number of molecules can be fabricated. The MCBJ and STM-BJ technique enables us to prepare the single-molecule junction. In this review, we focused on the rectification properties of the molecular diode. The rectification properties were initially proposed for the molecular junction, where the donor and acceptor units were connected via an insulating unit (Aviram-Ratner (A-R) model). Recent experimental studies revealed that the rectification properties appeared when the conduction orbital was strongly hybridized with one of the metal electrodes, in addition to the A-R type molecular diode. High rectification ratio of more than 100 was reported for the molecular diode based on the asymmetric metal-molecule coupling. Recent developments revealed that the structural degree of the freedom of the molecule(s), the surface morphology of the metal electrodes, and environment of the molecular junctions crucially affect the rectification properties of the molecularly thick junctions and the single-molecule junctions. The measurement technique of a small number of molecules has been improved in recent years. Currently, thermopower, force, atomic and electronic structures in the molecular junctions can be studied with advanced measurement techniques on the single molecule scale and link themselves to the performance and electronic function of devices. Precise control over the chemistry of the molecular backbone as well as the metal-molecular interface structures are crucial for understanding the unique electronic function of the molecular junctions, which could be an important aspect of fabricating future single-molecule-based electronic devices.
